# In-Depth Comparison of Lysine-Based Antibody-Drug Conjugates Prepared on Solid Support Versus in Solution

**DOI:** 10.3390/antib7010006

**Published:** 2018-01-07

**Authors:** Keith J. Arlotta, Aditya V. Gandhi, Hsiao-Nung Chen, Christine S. Nervig, John F. Carpenter, Shawn C. Owen

**Affiliations:** 1 Department of Bioengineering, University of Utah, Salt Lake City, UT 84112, USA; keith.arlotta@utah.edu; 2 Department of Pharmaceutical Sciences, Anschutz Medical Campus, University of Colorado, Aurora, CO 80045, USA; ADITYA.GANDHI@ucdenver.edu (A.V.G.); John.Carpenter@ucdenver.edu (J.F.C.); 3 Department of Pharmaceutics and Pharmaceutical Chemistry, University of Utah, Salt Lake City, UT 84112, USA; hsiao.chen@utah.edu; 4 Department of Medicinal Chemistry, University of Utah, Salt Lake City, UT 84112, USA; christine.nervig@utah.edu

**Keywords:** antibody drug conjugates, protein A, LC/MS, Raman, DSC, DLS, ITC, trastuzumab, DM1, one-step

## Abstract

Antibody drug conjugates are a rapidly growing form of targeted chemotherapeutics. As companies and researchers move to develop new antibody–drug conjugate (ADC) candidates, high-throughput methods will become increasingly common. Here we use advanced characterization techniques to assess two trastuzumab-DM1 (T-DM1) ADCs; one produced using Protein A immobilization and the other produced in solution. Following determination of payload site and distribution with liquid chromatography-mass spectrometry (LC/MS), thermal stability, heat-induced aggregation, tertiary structure, and binding affinity were characterized using differential scanning calorimetry (DSC), dynamic light scattering (DLS), Raman spectroscopy, and isothermal titration calorimetry (ITC), respectively. Small differences in the thermal stability of the C_H_2 domain of the antibody as well as aggregation onset temperatures were observed from DSC and DLS, respectively. However, no significant differences in secondary and tertiary structure were observed with Raman spectroscopy, or binding affinity as measured by ITC. Lysine-based ADC conjugation produces an innately heterogeneous population that can generate significant variability in the results of sensitive characterization techniques. Characterization of these ADCs indicated nominal differences in thermal stability but not in tertiary structure or binding affinity. Our results lead us to conclude that lysine-based ADCs synthesized following Protein A immobilization, common in small-scale conjugations, are highly similar to equivalent ADCs produced in larger scale, solution-based methods.

## 1. Introduction

The success of Kadcyla^®^ (Roche/Genentech, San Francisco, CA, USA) and Adcetris^®^ (Seattle Genetics, Seattle, WA, USA), as well as the recent approval of Besponsa^®^ (Pfizer/Wyeth Pharmaceuticals, Inc., Philadelphia, PA, USA) and reintroduction of Mylotarg^®^ (Pfizer Inc., New York, NY, USA), mark great strides in antibody–drug conjugate (ADC) technology [1]. Encouraging clinical results, along with the potential for greater financial returns [2], has led to a surge in interest with over 75 ADCs in clinical trials as of November 2017 [3,4]. Next generation ADCs are emerging with the incorporation of more stable linkers, higher drug-to-antibody ratios (DAR) and reduced levels of unconjugated antibody [5]. Although there has been a recent shift towards site-specific conjugation methods through engineered cysteines or enzymatic linkage, lysine conjugates still account for ~25% of ADCs currently in clinical trials [3].

**Figure 1:**
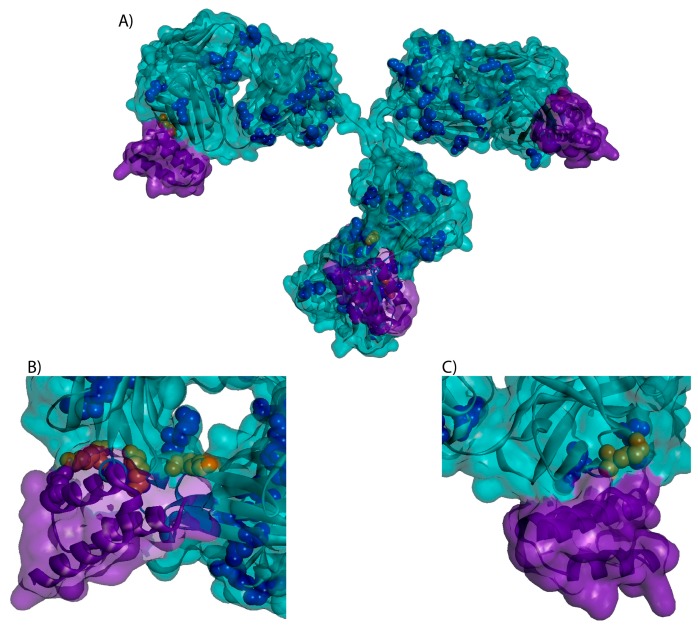
3-D structure of a trastuzumab (teal) bound to Protein A (purple) with lysines identified in blue. Four unique lysines (orange) were found to be within 10 Å of the protein A binding sites. Protein A has four potential binding sites on an IgG, two per heavy chain with one site in the Fab and one in the Fc region. (**A**) Full IgG (**B**) Fc region (**C**) Fab region.

As researchers work to develop and optimize new ADCs, high-throughput screening and conjugation methods, such as those described by Catcott et al. [6] and Puthenveetil et al. [7], have become increasingly attractive. In particular, the use of Protein A to immobilize monoclonal antibodies (mAbs) during drug conjugation is a potential technique for small-scale reactions. Protein A immobilization eliminates the need for buffer exchanges and simplifies the purification process, facilitating parallel ADC conjugations, increased throughput, and higher efficiency [8]. However, the potential interference of Protein A binding on lysine conjugation and subsequent drug distribution in ADCs has not been studied. Protein A can bind an IgG1 on the Fc and Fab regions for a total of four potential binding sites [9,10]. Based on inspection of crystal structures (PBD: 3D6G, 5U4Y, 4HKZ, 1IGT), there are four unique lysines within 10 Å of any Protein A binding site (H65, H252, H321, H342) as shown in Figure 1. As such, we sought to understand if Protein A binding to an antibody reduces payload conjugation at the proximal sites due to steric hindrance.

Dramatic improvements in physicochemical characterization methods since the first generation of ADCs were developed facilitate researcher’s ability to evaluate ADC stability, drug-loading, and binding characteristics [11]. Here we utilize peptide mapping techniques to analyze potential differences in drug distribution found between ADCs synthesized using solid support, Protein A magnetic agarose beads (On-Bead), and using traditional in solution conjugation (Off-Bead). Furthermore, we sought to evaluate if any changes in payload site distribution have a measurable effect on ADC characteristics (e.g., thermal stability, antigen binding) as measured by differential scanning calorimetry (DSC), dynamic light scattering (DLS), Raman spectroscopy, or isothermal titration calorimetry (ITC).

## 2. Results

### 2.1. ADC Synthesis

**Figure 2:**
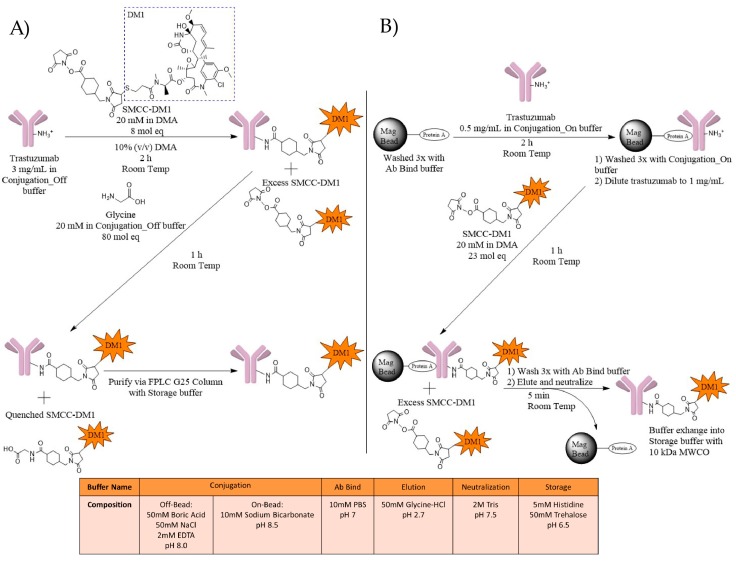
Schematic representation of the lysine-based conjugation of trastuzumab with a SMCC-DM1 payload using (**A**) Off-Bead and (**B**) On-Bead methods. (**A**) Trastuzumab at 3 mg/mL was mixed with 8 mol eq of 20 mM SMCC-DM1 in dimethylacetamide (DMA). Additional DMA was added to reach a 10% organic solution. Following a 2-h incubation at room temperature, 80 mol eq of 20 mM glycine in Conjugation_Off buffer (50 mM Boric Acid, 50 mM NaCl, 2 mM EDTA, pH 8.0) was added to quench unreacted SMCC-DM1. The solution was then purified using FPLC fractionation with a G25 column in Storage buffer; (**B**) Trastuzumab at 0.5 mg/mL was allowed to bind with pre-washed Protein A magnetic beads (1:2 µL beads:µg mAb) for 2 h at room temperature. Bound antibodies were washed with Conjugation_On buffer (10 mM Sodium Bicarbonate, pH 8.5) and reconstituted to 1.0 mg/mL. 23 mol eq of 20 mM SMCC-DM1 in DMA was added. The reaction was allowed to proceed for one hour at room temperature while mixing. The beads were washed to remove excess SMCC-DM1 and T-DM1 was eluted and neutralized. T-DM1 was then buffer exchanged into the Storage buffer using pre-wet 10 kDa MWCO centrifuge tubes.

Two conjugation methods, with (On-Bead) and without (Off-Bead) Protein A-coated magnetic beads (Figure 2), were used to evaluate the impact of antibody immobilization prior to conjugation on DAR, conjugation sites, and physical stability. Off-Bead samples were prepared through the reaction of trastuzumab with SMCC-DM1 for 2 h, followed by a one-hour incubation with glycine to quench any unreacted SMCC-DM1, and Fast Protein Liquid Chromatography (FPLC) purification to remove the excess SMCC-DM1 (Figure 2A). For On-Bead samples, trastuzumab was first immobilized using Protein A magnetic beads, followed by incubation with SMCC-DM1. After a one-hour incubation, the beads were washed and the trastuzumab-MCC-DM1 conjugate (T-DM1) was eluted and buffer exchanged into a histidine-trehalose storage buffer (Figure 2B). We first characterized ADCs by intact LC/MS to determine the drug distribution and average DAR. We defined the acceptable average DAR as DAR 3.5 ± 0.5 to approximate clinically relevant lysine-based ADCs such as Kadcyla^®^ (Roche/Genentech, San Francisco, CA, USA) (DAR 3.5). Further analysis of acceptable Off-Bead and On-Bead ADCs was used to measure important physicochemical characteristics.

### 2.2. Mass Spectrometry (MS)

#### 2.2.1. DAR Analysis

**Figure 3:**
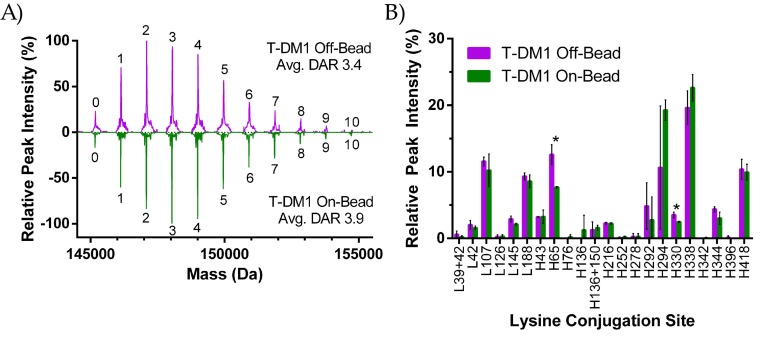
(**A**) Intact LC/MS analysis of deglycosylated T-DM1 Off-Bead (purple) and On-Bead (green). Integer values indicate the number of drugs per antibody found for that peak population as determined by the approximately 956 Da difference in mass between peaks. Average drug-to-antibody ratio (DAR) values are a weighted average of each DAR peak; (**B**) Relative peak intensity of conjugation sites following tryptic digestion for Off-Bead (purple) and On-Bead (green). An “L” denotes a lysine on the light chain while “H” indicates that the lysine is on the heavy chain. Samples were digested in triplicate from a single antibody–drug conjugate (ADC) batch for both Off-Bead and On-Bead prior to analysis. Conjugation sites are labeled on the x-axis with dual conjugated peptide sequences denoted with a “+” between the two sites. * indicates statistical significance between the conjugation site of Off-Bead and On-Bead (*p* ≤ 0.05). Standard deviation is represented by error bars above and below each data set.

Lysine-based ADC conjugation produces a heterogeneous population with a range of drug-to-antibody ratios (DAR) [12]. As shown in Figure 2, payload conjugation was achieved through a reaction between the linker NHS-Ester with solvent accessible lysines. We used intact LC/MS to analyze the drug-loading profile of each T-DM1 sample and quantify the average DAR as shown in the deconvoluted mass spectrum (Figure 3A). Prior to analysis, ADCs were deglycosylated with PNGase F to simplify the mass profile. The number above each peak represents the number of drugs attached to that ADC species, with the “0” peak representing unconjugated trastuzumab. Mass differences between peaks were approximately 956 Da which corresponds to the expected MW shift from the addition of a single SMCC-DM1 payload. As opposed to the 2-step conjugation method of Kadcyla, T-DM1 On-Bead and Off-Bead were prepared through one-step conjugation, resulting in no free-linker species. Each peak height represents the intensity of that DAR species relative to the highest intensity DAR peak. Shown here, both the On-Bead and Off-Bead samples have similar DAR distributions with equivalent minimum and maximum DAR (0 and 10 respectively) as well as a comparable unconjugated antibody (~4%) and average DAR as calculated by a weighted average of each species. Ionization efficiency was assumed to be equal among all DAR species.

#### 2.2.2. Peptide Mapping

Following reduction and alkylation of ADCs with dithiothreitol (DTT) and iodoacetamide (IAM) respectively, sequencing-grade trypsin was used to cleave mAbs at arginines and unconjugated lysines. Trypsin cleavage is highly efficient unless the lysine is conjugated or followed by a proline [13,14]. Nevertheless, while trypsin cleavage after a conjugated lysine is unlikely, we observed multiple instances of this occurring throughout several replicates. Following digestion, peptides were separated and analyzed by coupled LC-MS/MS. Using previously reported DM1 signatures (*m*/*z*: 547.221), extracted ion chromatography (XIC) was used to compare the relative abundance of drug conjugated peptides. XIC peak areas were normalized relative to a leu-enkephalin spiked peak for all samples (Table S1).

We identified 44 lysine conjugation sites between both samples. Among these commonly conjugated sites, only H65 and H330 were different in their conjugation abundance at a statistically significant level (Figure 3B). Of the 4 lysines identified as within 10 Å of a Protein A binding site (H65, H252, H321, H342), only H65 showed a relatively high (≥0.5% population) presence for either ADC. In the On-Bead sample, conjugation percentage of H65 was reduced by almost 40% relative to Off-Bead, strongly suggesting steric hinderance at this site. For non-common conjugation sites, Off-Bead had exclusive conjugation at the H396 site, while the On-Bead sample contained exclusive conjugation at the H76, H136, and H342 sites. It is worth noting that in all cases of conjugation site exclusivity, conjugations were only seen in 1 of the 3 replicates for the ADC in which conjugation was observed, which is indicative of the high level of heterogeneity within the sample. To further investigate potential interference between Protein A binding and DM1 conjugation, Off-Bead samples were split into three groups from a single batch: the first group was purified using Protein A after conjugation, the second group was exposed to the elution buffer and then neutralized, while the final group underwent no further purification. Peptide mapping data was collected and compared between the three groups (Figure S1). The elution process did not cause any statistically significant shifts in the DM1 conjugation site relative to untreated Off-Bead; however, significant shifts were seen between the Protein A purified sample and control samples at the L188 and H338 sites.

### 2.3. Dynamic Light Scattering (DLS)

**Figure 4:**
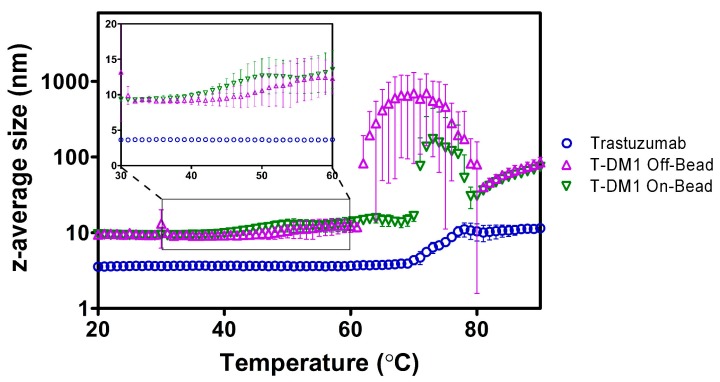
z-average size of trastuzumab (blue circle), Off-Bead (purple triangle), and On-Bead (green triangle) as a function of temperature measured by dynamic light scattering (DLS). Inlay shows the temperature of initial aggregation for Off-Bead and On-Bead. Data points represent average size ± standard deviation of triplicate values. Trastuzumab data was originally published in Gandhi et al. [16].

Using DLS, we characterized the heating-induced changes in protein aggregation by monitoring z-average size of the trastuzumab and ADC samples as a function of temperature [15]. The z-average size for both ADCs at the starting temperature of 20 °C was ~9.5 nm while trastuzumab had a much smaller initial size at ~3.5 nm (Figure 4). The T-DM1 Off-Bead sample had an aggregation onset temperature (T_onset_) of 57.3 ± 6.6 °C, while the On-Bead sample had a T_onset_ of 51.0 ± 7.1 °C. The On-Bead sample had a main aggregation event (T_agg_) occurring at 74.3 ± 2.4 °C compared to 65.0 ± 4.2 °C for Off-Bead. Trastuzumab had substantially higher T_onset_ and T_agg_ values at 70.7 ± 0.9 °C and 74.7 ± 1.2 °C, respectively. These results are consistent with previous reports that conjugation of hydrophobic payloads decreases the temperature of initial aggregation relative to the native antibody [16,17].

### 2.4. Differential Scanning Calorimetry (DSC)

**Figure 5:**
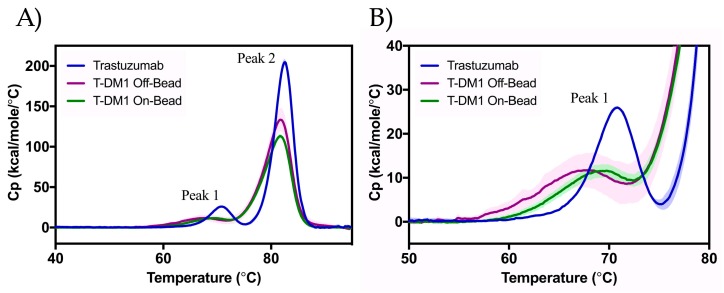
Thermal stability of trastuzumab (blue), Off-Bead (purple), and On-Bead (green) as assessed by DSC ranging from 40 °C to 90 °C (**A**) with the first melting point highlighted in (**B**). Peak 1 is indicative of to the unfolding of the C_H_2 domain, while Peak 2 corresponds to the unfolding of the Fab region and C_H_3 domain. Standard deviation is represented by the shading around each line. Trastuzumab data was originally published in Gandhi et al. [16].

DSC thermograms provide useful insight into the thermal stability of the ADCs related to denaturation (Figure 5). We observed two main transitions: Peak 1 corresponds to C_H_2 domain unfolding and Peak 2 corresponds to the unfolding of the Fab region and C_H_3 domain [18]. Statistically significant decreases in the midpoint temperature of thermal transitions (T_m_) were seen between trastuzumab and either ADC at both transitions (Table 1). The On-Bead and Off-Bead T-DM1 showed identical T_m,2_ values (−0.9 °C lower than that of trastuzumab). While the T_m,1_ value for the Off-Bead conjugate was significantly lower than that of the On-Bead sample, indicating greater stability of On-Bead in the C_H_2 domain.

### 2.5. Raman Spectroscopy

**Figure 6:**
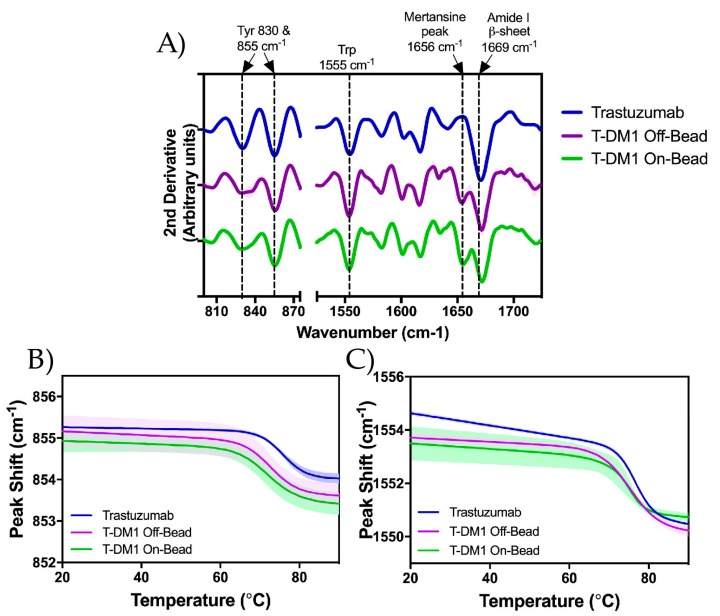
Analysis of secondary and tertiary structures of trastuzumab (blue), Off-Bead (purple) and On-Bead (green) as measured by Raman spectroscopy. (**A**) 2nd derivative Raman spectra measured at 20 °C from 800 to 1725 cm^−1^. Correlating structures are labeled above the figure with dotted lines at the respective wavenumber; (**B**,**C**) Thermally induced peak shifts in designated structures. Tyrosine 855 cm^−1^ in panel **B** and tryptophan 1555 cm^−1^ in panel **C**. Standard deviation is represented by the shading around each line. Trastuzumab data was originally published in Gandhi et al. [16].

We monitored the higher order structure of each sample using Raman spectroscopy at 20 °C. The correlation between various Raman band frequencies and their respective protein structures has been extensively characterized previously by Zai-Qing Wen [19]. The relevant Raman frequency bands and correlating structures are listed in Table 2. Distinct differences can be seen between the ADCs and trastuzumab only at the 1656 cm^−1^ frequency which has been correlated to spectral interference from the conjugated payload (DM1) (Figure 6A) [16]. The Raman spectrum of each sample was quantitatively compared using the weighted spectral difference (WSD) method described by Dinh et al. [20,21] relative to unmodified trastuzumab. Comparison ranges were defined using the trastuzumab spectrum around the structures identified in Table 2. The ranges around the tryosine and tryptophan side chains began at the wavenumbers in which the peak magnitude dropped below zero and ended when the magnitude values became positive; this incorporated the entire trough around each peak. As the DM1 interference was not present in the trastuzumab sample, the range was defined by the minima surrounding where the DM1 interference was seen in the ADC spectra. The final range was defined by the minima after the DM1 interference to the maxima following the β-sheet peak. The %WSD values can be found in Table S2. For the peaks at 830 cm^−1^ and 855 cm^−1^, roughly 2.5% and 0.5% spectral shifts were seen respectively, with a 1% shift seen at the tryptophan side chain (1555 cm^−1^). As expected, greater shifts were seen for both Off-Bead and On-bead samples at the 1656 cm^−1^ peak range, with %WSD values of roughly 10% and 12% respectively relative to trastuzumab. The relatively larger %WSD of On-Bead can be explained by the higher average DAR of the On-Bead corresponding to an increased interference from DM1. Additionally, a spectral difference of ~4% was calculated for both ADCs in the 1671–1697 cm^−1^ range which incorporates the remainder of the Amide I region. The relatively small spectral differences seen in each region, indicate that DM1 conjugation does not significantly affect the tertiary structure of the antibody scaffold.

To further assess tertiary structure as a function of temperature, we monitored the peak shifts of tertiary structural markers; tyrosine, at 855 cm^−1^ (Figure 6B), and tryptophan, at 1555 cm^−1^ (Figure 6C), side chains were monitored while increasing temperature step-wise from 20 to 90 °C. The 855 cm^−1^ marker shifted down by approximately 1.5 cm^−1^, while the 1555 cm^−1^ peak shifted down approximately 3 cm^−1^. In both cases, there was no statistically significant difference in peak shift or midpoint temperatures (T_mid_) between On-Bead and Off-Bead samples. T_mid_ values at 855 cm^−1^ and 1555 cm^−1^ were approximately 71.8 °C and 74.5 °C for Off-Bead, 71.5 °C and 73.5 °C for On-Bead and 75.4 °C and 74.8 °C for trastuzumab, respectively. Additionally, no statistically significant difference in the onset temperature (T_initial_) was calculated between Off-Bead and On-Bead at the 855 cm^−1^ wavenumber (68.7 °C and 67.3 °C respectively) or 1555 cm^−1^ peak at 71.0 °C and 71.7 °C respectively. Trastuzumab had a significantly higher T_initial_ at 855 cm^−1^ of 72.3 °C and a non-significantly greater T_initial_ of 73.8 °C at 1555 cm^−1^.

### 2.6. Isothermal Titration Calorimetry (ITC)

**Figure 7:**
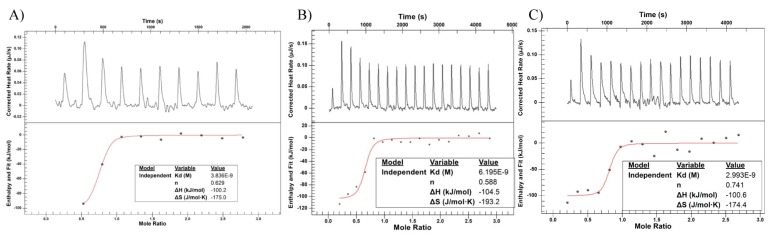
Binding data for (**A**) trastuzumab (**B**) Off-Bead and (**C**) On-Bead against recombinantly produced HER2. Raw data for heat produced after titration is found in the top panels while the fitted data is found in the bottom panels.

We utilized ITC to measure the driving forces of the interaction between the antibody and the target receptor (HER2). The change in heat data is fit to determine the stoichiometry of the interaction (n), the disassociation constant (K_d_), total change in enthalpy (ΔH) and change in entropy (ΔS) [22]. ITC samples were run in duplicate with representative graphs shown in Figure 7. Calculated average values for n, K_d_, ΔH, and ΔS are shown in Table 3. We detect no significant difference in the calculated stoichiometry and K_d_ calculations are consistent with previously reported values in the literature [23,24,25]. As such, DM1 conjugation through either method does not affect the binding affinity of trastuzumab. Likewise, changes in enthalpy and entropy as a result of binding are not significantly different between samples.

## 3. Discussion

### 3.1. ADC Synthesis and Payload Distribution

LC/MS provides significantly more information about the drug distribution of an ADC than was previously possible using UV-Vis. While UV-Vis can be used to determine an average DAR, other details such as the maximum DAR or the level of unconjugated antibody cannot be resolved [11,12,26]. Such a limitation was highlighted in the case of Mylotarg^®^ (Pfizer Inc., New York, NY, USA) which had an average DAR of 3, similar to that of Kadcyla^®^ (Roche/Genentech, San Francisco, CA, USA) (DAR 3.5); however, ~50% of the antibodies were unconjugated [11]. The dichotomy in product distribution resulted in drastically different pharmacokinetic profiles and variable efficacy within a single dose [27,28]. In contrast, LC/MS spectra reveal the heterogeneity of an ADC population in respect to relative drug distribution, average DAR, maximum DAR, and percent unconjugated antibody.

Using LC/MS, we show that both On-Bead and Off-Bead ADC conjugates contain similar drug distributions, with equivalent maximum DAR and levels of unconjugated antibody. On-Bead samples in this case had a higher average DAR, which correlates to a larger fraction of ‘high DAR’ species. It is interesting to note that the On-Bead conjugation required higher equivalents of payload to achieve the same average DAR. In the large-scale conjugations used to produce the ADCs for characterization and analysis, 8 eq of SMCC-DM1 were used to achieve average DAR 3.4 for the Off-Bead sample, while 23 eq of SMCC-DM1 were needed for an average DAR 3.9 for the On-Bead sample. We conclude that translating from one conjugation method to the other will require an adjustment in reaction conditions. Additional testing is needed to establish if there is a direct relationship between payload equivalents needed to achieve the same DAR between conjugation methods.

We further utilize LC/MS to deduce the exact conjugation locations on the parent antibody. Reduction and trypsinization of the ADC produces smaller peptides that are matched to known molecular weights. For lysine-based ADCs, DM1 conjugation prevents cleavage after the conjugated lysine and proportionally modifies the weight of the peptide. Trastuzumab contains 92 potential amine conjugation sites (88 lysines + 4 N-Termini) [13]. We identified 44 conjugation sites which is less than the 82 sites previously reported using this peptide mapping method [13]. Potentially, these differences are a result of incomplete ionization of the antibody or a higher limit-of-detection.

Lysine based conjugation is an innately heterogeneous process with an average batch potentially containing over 4.5 million unique conjugates [13]. Of the 4 lysine residues identified as being proximal (≤10 Å) to a Protein A binding site, H65 was the only residue with significant (≥0.5%) levels of conjugation in either ADC. A significant decrease (40%) occurred in conjugation at the H65 site in On-Bead relative to Off-Bead, strongly suggesting steric hindrance at this site caused by Protein A immobilization. H330, located in the hinge region of the Fc, was also subject to a statistically significant decrease in the On-Bead sample. We posit that this could be due to conformational shifts in the hinge region of trastuzumab when bound to Protein A. While these differences in conjugation site distribution did not ultimately have a significant effect on the measured characteristics, likely due to the highly heterogeneous product of lysine-based ADCs, this reveals potential concerns for the development of site-specific conjugates. We have shown here that Protein A can have an inhibitory effect near the binding site and as such, design of site-specific antibodies should avoid engineering of sites proximal to the Protein A binding domains.

Investigation into Protein A purification of T-DM1 conjugates did not reveal any significant decreases in ADCs containing conjugation at proximal lysines. This can be explained by the multiple binding domains of Protein A on an IgG. Since there are 4 potential Protein A binding sites, conjugation would likely have to occur at multiple sites in order to significantly block binding. Nevertheless, the population of two non-proximal conjugation sites, L188 and H338, did decrease to a statistically significant extent following Protein A purification. Both of these sites were unaffected by Protein A binding prior to conjugation, yet purification of the complete ADC with Protein A resulted in 22% and 20% decreases in population respectively. The cause of these decreases is not readily inferred by the data or modeling based on Figure 1. As such, we suggest that when developing a specific ADC, a similar analysis should be attempted prior to using Protein A for purification or collection to ensure complete recovery. Further, a high level of variance was seen between replicate digestions and analysis within an ADC batch, suggesting that measured differences in conjugation site distribution may decrease with an increased number of replicates.

### 3.2. Thermal Stability and Aggregation

We assess thermal stability as a result of denaturation using DSC. Consistent with previous findings, two endothermic peaks are seen corresponding to the unfolding of the C_H_2 domain of the Fc region (T_m,1_) and the C_H_3 domain and Fab region (T_m,2_) [18]. The correlation of each peak with its reported region is supported by the lower change in enthalpy of Peak 1 relative to Peak 2 [17]. As expected, DM1 conjugation decreased T_m,1_ and T_m,2_ values with statistically significant decreases in T_m,1_ for all samples, and between trastuzumab and either ADC for T_m,2_. Our reported melting temperatures are consistent with previously reported values [13,22]. Both Off-Bead and On-Bead showed a statistically significant 0.9 °C decrease in T_m,2_ relative to trastuzumab. The Off-Bead T_m,1_ value is also consistent with previous reports at a decrease of 2.9 °C compared to trastuzumab; however, On-Bead T_m,1_ only decreased 1.0 °C relative to trastuzumab, implying greater thermal stability in the C_H_2 region.

We monitored thermally induced aggregation vis-à-vis colloidal stability using DLS as shown in Figure 4. As expected from previous reports [17], both ADCs show substantial decreases in thermal aggregation onset temperature relative to the parent antibody. The differences in onset and main aggregation temperatures between ADCs poses an interesting question. As shown in Figure 4, aggregation trends of the ADC samples are contradictory—the T_onset_ of On-Bead is lower than that of Off-Bead, yet the corresponding T_agg_ values are reversed. Nevertheless, the differences in T_onset_ and T_agg_ were not statistically significant. The large variability of T_onset_ values for Off-Bead and On-Bead can be explained by the highly heterogenous ADC samples, as supported by the much smaller standard deviations calculated for unmodified trastuzumab. While the differences in T_onset_ are not statistically significant, the decrease in T_onset_ of On-Bead can be explained by the slightly higher average DAR. Adem et al. characterized the aggregation of thiol conjugated ADCs separated by DAR and concluded that high DAR species are significantly more susceptible to aggregation [29]. Overall, our data supports previous reports that conjugation of a hydrophobic payload decreases thermal stability and promotes aggregation at lower temperatures relative to the parent antibody [13,17,22].

### 3.3. Higher Order Structure and Binding Affinity

Raman Spectroscopy was used to analyze whether DM1 conjugation through either Off-Bead or On-Bead synthesis methods effects the higher order structure of ADCs relative to the parent antibody. With the exception of the DM1 interference at 1656 cm^−1^ as described by Gandhi et al. [16], little difference was seen between the Raman spectra of the trastuzumab and either Off-Bead or On-Bead sample. The overall Raman spectra was divided into 5 regions correlating to different antibody structures (Table 2). Quantitative analysis between the spectra was done using the weighted spectral difference method relative to unmodified trastuzumab as described by Dinh et al. [20] (Table S2). The similarity of the Raman spectra (Figure 6A) indicates that the use of Protein A to immobilize trastuzumab prior to conjugation did not affect the tertiary structure of the final product, nor does DM1 conjugation significantly affect the tertiary structure of trastuzumab. To further probe tertiary structure as a function of temperature, we investigated two wavenumbers, 855 cm^−1^ and 1555 cm^−1^ corresponding to tyrosine and tryptophan respectively, while increasing temperature from 20 to 90 °C. Consistent with trends from our DLS and DSC data, hydrophobic payload conjugation significantly decreased the thermal stability of the ADC tertiary structure as measured by T_initial_ and T_mid_ values.

Antibody-antigen binding is a product of noncovalent interactions including electrostatic, van der Waals, hydrogen bonds, and hydrophobic effects [30]. ITC is a useful tool to investigate the thermodynamics controlling an antibody-antigen interaction and calculate crucial parameters such as the K_d_, stoichiometry, ΔH, and ΔS [31]. Previous reports show that DM1 conjugation does not significantly affect the binding affinity (measured here as K_d_) of trastuzumab [23,24,25]. Using ITC, we confirmed this finding with calculated K_d_ values of approximately 5 nM for all samples [23,24,25]. Stoichiometry, ΔH, and ΔS were also found to be equivalent between all samples. ITC provided an important surrogate metric for efficacy that could not be obtained from the other characterization techniques used such as DSC, DLS, and Raman. The consistency and similarity of the K_d_ value found for both ADCs indicates that, despite minor differences in thermal stability, ADC binding was not affected by On-Bead or Off-Bead conjugation. Therefore, differences in thermal stability can likely be explained by inherent heterogeneity in the lysine-based conjugation process and not a direct result of On-Bead conjugation.

## 4. Materials and Methods

### 4.1. Synthesis

#### 4.1.1. T-DM1 Off-Bead Synthesis

Trastuzumab (Genentech, South San Francisco, CA, USA) was dialyzed against borate buffer (50 mM Boric Acid, 50 mM NaCl, 2 mM EDTA, pH 8.0) using 10 kDa MWCO Slide-A-Lyzer Dialysis Cassettes (ThermoFisher Scientific, Waltham, MA, USA) to a final concentration of 3 mg/mL. SMCC-DM1 (ALB Technology Limited, Hong Kong, China) was prepared to 20 mM in dimethylacetamide (DMA). SMCC-DM1 (8 mol eq) was added to the trastuzumab (25 mg) slowly while stirring. Additional DMA was added to the trastuzumab solution such that the final organic ratio was 10% *v*/*v*. The reaction was allowed to proceed for 2 h at room temperature (RT). Glycine (20 mM, 80 mol eq) in borate buffer was added for 1 h at RT to quench excess SMCC-DM1. T-DM1 Off-bead was purified using FPLC fractionation with a Sephadex G25 Fine (GE Healthcare Life Sciences, Marlborough, MA, USA) column in histidine buffer (5 mM histidine, 20 mg/mL trehalose, pH 6.0).

#### 4.1.2. T-DM1 On-Bead Synthesis

Trastuzumab (Genentech, South San Francisco, CA, USA) was dialyzed against borate buffer (50 mM Boric Acid, 50 mM NaCl, 2 mM EDTA, pH 8.0) using 10 kDa MWCO Slide-A-Lyzer Dialysis Cassettes (ThermoFisher Scientific, Waltham, MA, USA) to a final concentration of 0.5 mg/mL. All wash steps were repeated for a total of 2 washes. Using a magnetic separation stand, Magne^®^ Protein A Beads 20% Slurry (Promega, Madison, WI, USA) were washed with Ab bind buffer (10 mM phosphate buffer, pH 7.0). 0.5 mg/mL trastuzumab (25 mg) was allowed to mix with the washed Protein A Beads (1:2 µL beads:ug mAb) for 2 h at RT. Bound trastuzumab was then washed with Ab bind buffer followed by amine conjugation buffer (10 mM sodium bicarbonate buffer, pH 8.5). Amine conjugation buffer was added to the initial reaction volume. SMCC-DM1 was prepared to 20 mM in DMA. SMCC-DM1 (23 mol eq) was added to the bound trastuzumab slowly and then vortexed. The reaction was allowed to proceed for 1 h at RT while mixing. Using the magnetic stand, the supernatant was removed and the bound T-DM1 ADC was washed with Ab bind buffer. Elution buffer (50 mM glycine-HCL, pH 2.7) was added to the original reaction volume and allowed to mix at RT for 5 min. After 5 min, the supernatant was removed and transferred to a new tube containing neutralization buffer (2M tris buffer, pH 7.5) at a 1:20 volumetric ratio. The eluted T-DM1 On-bead was then buffer exchanged into histidine buffer using 10 kDa MWCO Sartorious^®^ Vivaspin^®^ 20 centrifugal concentrators (FischerScientific, Waltham, MA, USA).

### 4.2. Mass Spectrometry

#### 4.2.1. DAR Analysis by LC/MS

Prior to mass spec (MS) analysis, 2 µL of PNGase F (New England Biolabs, Ipswich, MA, USA) was added to 100 µg T-DM1 (≥1 mg/mL) and incubated overnight at 37 °C to deglycosylate the IgG. Following deglycosylation, the sample was buffer exchanged into ammonium acetate buffer (50 mM ammonium acetate, pH 7.0) using a 10 kDa Amicon Ultra 0.5mL centrifugal filter (EMD Millipore, Burlington, MA, USA). Intact protein LC/MS analysis was performed on an ACQUITY UPLC I-Class with a Xevo G2-S QToF mass spectrometer (Waters Corp., Milford, MA, USA). Deglycosylated ADC samples were further desalted with a MassPREP micro desalting column (Waters, Milford, MA, USA) using a 6-min linear gradient run at flow rate of 0.3 mL/min, 80 °C. The gradient was programmed as follows: 5% B from 0 to 2 min, 5–90% B from 2 to 5 min, then 90–5% B from 5 to 6 min. The mobile phase A was water with 0.1% formic acid and the mobile phase B was acetonitrile with 0.1% formic acid. The mass spectrometer was operated in positive electrospray ionization (ESI) mode. The capillary voltage was 3 kV and the sampling cone voltage was 150 V. The source temperature was 150 °C and the desolvation temperature was 500 °C. The source desolvation gas flow and cone gas flow was 800 L/h and 10 L/h, respectively. The recorded mass spectra were combined and deconvoluted using MassLynx 4.1 (Waters, Milford, MA, USA)

DAR for each species peak was identified by dividing the difference between the MW of the peak and the unmodified trastuzumab peak (~145 kDa) by the expected MW of the payload/linker species (956 Da). The following analysis of MS results assumes equal ionization efficiencies between various DAR species of the ADC. The relative peak intensity of each DAR species was calculated by dividing the peak area of that species by the total peak area of that sample. Average DAR for each ADC was calculated by summing the product of each DAR species multiplied but it’s relative peak intensity within a sample.

(1) $$\mathrm{Avg}\;\mathrm{DAR}=\overset{\mathrm{DAR}\;\max}{\underset{i=\mathrm{DAR}\;\min}{\sum }}({\mathrm{DAR}}_{i}\times {\mathrm{Relative}\;\mathrm{Peak}\;\mathrm{Intensity}}_{{\mathrm{DAR}}_{i}})=\overset{\mathrm{DAR}\;\max}{\underset{i=\mathrm{DAR}\;\min}{\sum }}({\mathrm{DAR}}_{i}\times \frac{{\mathrm{Peak}\;\mathrm{Area}}_{{\mathrm{DAR}}_{i}}}{\mathrm{Total}\;\mathrm{Peak}\;\mathrm{Area}})$$

#### 4.2.2. Peptide Mapping

The digestion procedure was modified from the protocol described by Agilent Technologies [32]. 20 μL of each ADC was prepared at 3 mg/mL. 20 μL of 10mM ammonium bicarbonate (pH 8.0), 50 μL of trifluoroethanol (TFE) diluted 1:10 in Millipore H_2_O, and 5 μL of 20 mM dithiothreitol (DTT), were added to the ADC. The mixture was allowed to react at 65 °C for 30 min. The mixture was then cooled to RT before adding 20 μL of 20 mM iodoacetamide (IAM) and then allowed to react for 1 h at RT in the dark. 5 μL of 20 mM DTT was added and allowed to react for 1 h at RT to quench excess IAM. The following was then added to the mixture: 200 μL 10 mM ammonium bicarbonate, 600 μL Millipore H_2_O, 40 μL of 0.1 mg/mL sequencing grade modified trypsin (Promega, Madison, WI, USA), the mixture was then incubated overnight at 37 °C while shaking at 300 rpm. 5 μL of formic acid diluted 1:10 in Millipore H_2_O was added to quench the digestion. The mixture was then lyophilized.

To perform peptide mapping analysis, 65 µg of the lyophilized, trypsin digested ADC samples were dissolved in 50 µL solvent containing 95% of water, 5% acetonitrile, and 0.1% formic acid. Then, 1 ng of leucine enkephalin (leu-enk, sequence: YGGFL) was added to the sample as an internal standard. 5 µL (6.5 µg) of the peptide mapping sample was injected for each LC run. The peptide mapping sample was analyzed by an ACQUITY UPLC I-Class coupled with a Xevo G2-S QToF mass spectrometer (Waters, Milford, MA, USA). The digested peptides were separated by an ACQUITY UPLC peptide CSH C18 column (2.1 mm × 100 mm, 1.7 µm particle size; Waters, Milford, MA, USA) using a linear gradient (5–42% B) at a flow rate of 0.2 mL/min over 55 min while maintaining 40 °C column temperature. Mobile phase A was water with 0.1% formic acid and mobile phase B was acetonitrile with 0.1% formic acid. The LC-MS/MS data was acquired by operating the mass spectrometer at MSE mode. The MSE mode configures the mass spectrometer to switch between low energy scans and high energy scans to generate intact peptide masses and fragmented peptide masses. At high energy scans, a collision energy ramp was set between 20 V and 50 V to fragment peptides. The ESI source was maintained at 150 °C. The capillary voltage is 3 kV and the sampling corn voltage is at 35 V. The cone gas flow is 5 L/h and the desolvation gas flow and temperature is 600 L/h and 350 °C. A mass signal (*m*/*z*: 556.277) from a continuously infused leucine enkephalin standard through the lockmass channel was used to provide the external mass calibration.

The acquired LC-MS/MS data was processed using BiopharmaLynx software (Waters, Milford, MA, USA). The lysine sidechain MCC-DM1 conjugation (+956.3644 Da) was set as a variable modification. The previously reported DM1 signature fragment ion (*m*/*z*: 547.221) was used to identify the drug conjugated peptides [13]. Extracted ion chromatographic (XIC) peaks of the drug conjugated peptides were used to compare the abundance of conjugation. The spiked leu-enk XIC peak area was used to normalizing all the drug conjugated peptide peak areas. Relative peak intensity of each conjugation site was determined using the same method described for DAR analysis with the normalized peak areas. Ionization efficiency of all peptides was assumed to be equal for our calculations; however, this is unlikely to accurately represent the ionization of each peptide fragment, thus the calculated relative peak intensities do not necessarily correlate with the relative abundance of each fragment.

### 4.3. Sample Prep

#### 4.3.1. Post-Conjugation Protein A Purification Study

T-DM1 was synthesized according to the Off-Bead protocol and purified using FPLC fractionation as described. Subsequent purification using Magne^®^ Protein A Beads 20% Slurry (Promega, Madison, WI, USA) was performed. Using a magnetic separation stand, Magne^®^ Protein A Beads 20% Slurry were washed with Ab bind buffer (10 mM phosphate buffer, pH 7.0). 0.2 mg/mL T-DM1 was allowed to mix with the washed Protein A beads (1:2 µL beads: µg mAb) for 1 h at RT. Using the magnetic stand, the supernatant was removed. Elution buffer (50 mM glycine-HCL, pH 2.7) was added (1:2 µL beads: µL elution buffer) and allowed to mix at RT for 5 min. After 5 min, the supernatant was removed and transferred to a new tube containing neutralization buffer (2M tris buffer, pH 7.5) at a 1:20 volumetric ratio. The eluted T-DM1 was then buffer exchanged into histidine buffer using 10 kDa MWCO Sartorious^®^ Vivaspin^®^ 20 centrifugal concentrators (FischerScientific, Waltham, MA, USA).

To control for the effect of elution buffer, a separate aliquot of T-DM1 was treated under similar conditions. T-DM1 was added to a 10 kDa MWCO Sartorious^®^ Vivaspin^®^ 20 centrifugal concentrator (FischerScientific, Waltham, MA, USA) and concentrated to a minimal volume through centrifugation at 12,000 rpm for 10 min. Elution buffer was added to the T-DM1 to 1 mg/mL and let stand at RT for 5 min. After 5 min, neutralization buffer at a 1:20 volumetric ratio was added. The T-DM1 was then buffer exchanged into histidine buffer.

#### 4.3.2. Characterization

Off-Bead and On-Bead T-DM1 samples were buffer exchanged using three repetitive cycles of concentrating and then diluting with the formulation buffer (5 mM histidine, pH 6.0 with 2% *w*/*v* trehalose and 0.009% *w*/*v* polysorbate-20) in an Amicon^®^ Ultra centrifugal filter with a 10-kDa molecular weight cut-off. All solutions were filtered using a pre-wetted 0.22 μm PVDF syringe filter (EMD Millipore, Burlington, MA, USA) before analyses.

### 4.4. Concomitant Raman Spectroscopy and DLS

First, the effect of drug conjugation on the structure of mAb was evaluated by collecting Raman spectrum of Off-Bead and On-Bead T-DM1 at 20°C using the Zetasizer Nano ZS (Malvern Instruments, Westborough, MA, USA) combined with a Kaiser Raman RxN1 spectrometer (Kaiser Optical Systems, Ann Arbor, MI, USA). 60 μL of sample at a protein concentration of ~12 mg/mL was pipetted into a 50-μL cuvette, sealed with a Teflon stopper and the Raman spectra were collected with 12-s exposure time and 20 co-additions. The formulation buffer without the protein was used to acquire a background Raman spectrum while keeping the same data acquisition parameters.

Additionally, the effect of heating on protein secondary and tertiary structure (Raman spectroscopy) as well as aggregation of Off-Bead and On-Bead T-DM1 samples was studied using the temperature-control capabilities of the Zetasizer Nano ZS. Raman and DLS data were collected sequentially by heating samples from 20 to 90 °C at every 1 °C intervals as previously described [16]. Raman spectra of the formulation buffer without the protein were also collected using the same temperature ramping protocol. For DLS data at each temperature, backscattering at 173° was recorded using the automated attenuator. Z-average size from DLS was plotted as a function of temperature to study heating-induced aggregation of Off-bead and On-bead T-DM1. Data analysis of Raman spectra was performed in the Helix Analyze Version 1.0.3 (Malvern Instruments Ltd., Westborough, MA, USA) as detailed out by Gandhi et al. [16]. T_onset_ is defined as the temperature at which the z-average size exceeded the initial size by 25% were the initial size was the average value of the first 5 temperature measurements. T_agg_ is defined as the temperature at which the z-average size exceeded the initial size by 100%. Percent weighted spectral difference (%WSD) was calculated using the following equation as described by Dinh et al. [20]. Where *n* is the number of measurements used, *y* is the magnitude at each wavenumber, and *A* and *B* refer to the reference and test sample respectively. %WSD values were calculated for each replicate of the Raman spectra using trastuzumab as the reference. Final values were reported as averages ± standard deviation in Table S2.

(2) $$\text{\%}\mathrm{WSD}=\frac{\sqrt{\sum_{i=1}^{n}\left[\left(\frac{1}{n}\right)\left(\frac{\left|y_{\mathrm{Ai}}\right|}{{\left|y_{A}\right|}_{\mathrm{Ave}.}}\right){\left(y_{\mathrm{Ai}}-y_{\mathrm{Bi}}\right)}^{2}\right]}}{\left|\sum_{i=1}^{n}y_{\mathrm{Ai}}\right|}$$

### 4.5. DSC

Thermal analysis of Off-Bead and On-Bead T-DM1 was performed using the Malvern MicroCal VP-Capillary DSC (Malvern Instruments, Westborough, MA, USA). All samples were analyzed at a protein concentration of 0.5 mg/mL in the formulation buffer. 550 μL of samples and buffer were added in a polypropylene round bottom 96-well plate (Microliter Analytical Supplies Inc., Suwanee, GA, USA), covered with a MicroMat^TM^ clear silicone mat (Thermo Scientific, Waltham, MA, USA) and placed in a temperature controlled storage box at 4 °C for the duration of analysis. The excess heat capacity was monitored across a scan range of 15–95 °C at a heating rate of 60 °C/h. Data were buffer subtracted and baseline corrected in the MicroCal Origin 7.0 software (Malvern Instruments, Westborough, MA, USA) and the temperature corresponding to the apex of endothermic transitions was defined as apparent T_m_.

### 4.6. ITC

Titrations were performed in an Affinity ITC LV (TA Instruments, Lindon, UT, USA) with a cell volume of 190 µL. The cell was loaded with 250–300 µL of HER2 (Speed Biosystems, Gaithersburg, MD, USA) at 0.8 to 1.2 µM and the syringe was loaded with the different preparations of trastuzumab at concentrations from 10 to 14 µM. The exact concentrations for each assay were used in the data analysis. During titration, the instrument was stirred at 100 rpm. Prior to starting data collection, the calorimeter was equilibrated to a baseline with a drift of less than 10 nW and standard deviation less than 100 µW/h over a five-min period. Prior to the first injection, a 60-s baseline was collected before the first injection of 0.2 µL the remaining injections that were used in the fitting were 2.5 µL delivered every 200 s. The reference cell was filled with 300 µL of nano-pure water. All data was collected with exothermic events up.

### 4.7. Statistics

All statistical analyses were performed using Microsoft Excel 2016 (Microsoft, Redmond, WA, USA) and GraphPad Prism version 7.00 for Windows (GraphPad Software, San Diego, CA, USA). Differences among pairs were assessed using Student’s *t*-test in Excel. Groups were assessed by one-way ANOVA with Bonferroni *post hoc* correction to identify statistical differences among three or more treatments in GraphPad Prism. Alpha levels were set at 0.05 and a *p*-value of ≤0.05 was set as the criteria for statistical significance. All data are presented as mean ± standard deviation.

### 4.8. 3-D Trastuzumab-Protein A Model

While a full IgG structure of trastuzumab bound to Protein A is not available, crystal structures of the trastuzumab Fc and Protein A (B-domain) bound to the trastuzumab Fab have been deposited in the RCSB Protein Data Bank. To create a model of the full trastuzumab IgG structure bound by Protein A at all 4 sites, available crystal structures were superimposed using the tether method in Biovia Discovery Studio Visualizer (v17.2.0.16349, Accelrys, San Diego, CA, USA). PDB 1IGT was used as the full-length IgG scaffold onto which the crystal structures of each trastuzumab fragment were superimposed. The trastuzumab Fab bound to Protein A (PDB 4HKZ), trastuzumab Fc (PDB 3D6G), and Fc-bound Protein A (PDB 5U4Y) were each aligned by tethering to the alpha carbons of the cysteine residues in their respective regions on PDB 1IGT. Using the resulting model, all trastuzumab lysine residues within 10 Å of a Protein A were identified.

## 5. Conclusions

Antibody-drug conjugates are complex macromolecules. Minor changes in a synthesis may propagate into major changes to physicochemical characteristics. As such, it is important to monitor several attributes with various analytical techniques to assess the impact of any changes. LC/MS is useful in determining DAR and drug distribution; DSC and DLS are useful in revealing perturbations in thermal stability; Raman spectroscopy is effective in detecting changes in tertiary structure and; ITC is suitable to measure binding attributes. Employing these techniques to compare an On-Bead versus Off-Bead synthetic approach to construct lysine-conjugated ADCs reveals nominal differences in drug distribution, which do not appear to impart significant differences in structure or function. Therefore, high-throughput or small-scale conjugation approaches using solid state resins to synthesize and evaluate ADCs are predictive of ADCs synthesized in larger scale, solution based methods.

## Figures and Tables

**Table 1:** Transition melting temperatures as calculated from differential scanning calorimetry (DSC) data. Comparison between Off-Bead and On-Bead showed a statistically significant difference (*p* ≤ 0.05) for only the T_m,1_ value. Data shown as an average ± standard deviation of triplicate measurements.

	T_m,1_ (°C)	T_m,2_ (°C)
Trastuzumab	70.6 ± 0.1	82.6 ± 0.1
T-DM1 Off-Bead	67.7 ± 0.3	81.7 ± 0.0
T-DM1 On-Bead	69.6 ± 0.3	81.7 ± 0.0

**Table 2:** Raman band frequency with correlated structure.

Raman Spectra (cm^−1^)	Structure
830 & 855	Tyrosine Side Chain
1555	Tryptophan Side Chain
1656	DM1
1669	β-sheet
1650–1680	Amide I

**Table 3:** Calculated values for the dissociation constant (Kd), stoichiometry (*n*), enthalpy (ΔH), and entropy (ΔS) from the fitted data in Figure 7. No statistical significance was seen between any of the calculated values (*p* ≤ 0.05). Data shown as an average ± standard deviation of duplicate measurements.

	K_d_ (nM)	*n*	ΔH (kJ/mol)	ΔS (J/mol × K)
Trastuzumab	5.56 ± 0.64	1.40 ± 0.77	−101.10 ± 5.10	−181.05 ± 16.15
T-DM1 Off-Bead	4.13 ± 2.06	0.64 ± 0.05	−102.60 ± 1.90	−182.25 ± 10.95
T-DM1 On-Bead	5.24 ± 2.25	0.66 ± 0.09	−106.10 ± 5.50	−196.50 ± 22.10

## Supplementary Materials

The following are available online at http://www.mdpi.com/2073-4468/7/1/6/s1, Table S1: Peptide Mapping Sequence List, Figure S1: ADC Purification with Protein A Magnetic Beads, Table S2: %WSD Data Table.
