# Supplementary material for: In-Depth Comparison of Lysine-Based Antibody-Drug Conjugates Prepared on Solid Support Versus in Solution
Source: Antibodies (Basel). 2018 Jan 7;7(1):6. doi: 10.3390/antib7010006 (PMC6698837; doi:10.3390/antib7010006)
Supplement: Supplementary file 1 [file antibodies-07-00006-s001.pdf]

Supplemental Material

Table S1: Peptide Mapping Sequence List
Figure S1: ADC Purification with Protein A Magnetic Beads
Table S2: %WSD Data Table

Table S1: LC/MS analysis of trypsin digested ADCs revealed 44 conjugated lysines. The sequence corresponding to each conjugated lysine as well as the respective population percentage (%) and standard deviation for each site are shown below. Peptide mapping samples were digested and analyzed in triplicate.

	Conjugated Lysine	Sequence	Population Percentage ± SD (%) Off-Bead	On-Bead
Light Chain	L39+42	VTITCRASQDVNTAVAWYQQKPGK	0.6 ± 0.4	0.2 ± 0.2
	L42	ASQDVNTAVAWYQQKPGKAPK	2.1 ± 0.5	1.6 ± 0.2
	L107	VEIKR	11.6 ± 0.5	10.2 ± 2.0
	L126	TVAAPSVFIFPPSDEQLK	0.2 ± 0.2	0.3 ± 0.2
	L145	EAKVQWK	2.9 ± 0.4	2.1 ± 0.1
	L188	ADYEKHK	9.3 ± 0.4	8.6 ± 0.8
Heavy Chain	H43	QAPGKGLEWVAR	3.2 ± 0.0	3.3 ± 0.8
	H65	YADSVKGR	12.6 ± 1.2	7.7 ± 0.1
	H76	FTISADTSKNTAYLQMNSLR	0.0 ± 0.0	0.2 ± 0.3
	H136	GPSVFPLAPSSKST	0.0 ± 0.0	1.3 ± 1.8
	H136+150	GPSVFPLAPSSKSTSGGTAALGCLVK	1.3 ± 1.0	1.6 ± 0.3
	H216	VDKK	2.3 ± 0.1	2.2 ± 0.1
	H252	THTCPPCPAPELLGGPSVFLFPPKPKDTLMISR	0.1 ± 0.1	0.2 ± 0.1
	H278	DTLMISRTPEVTCVVVDVSHEDPEVK	0.3 ± 0.4	0.3 ± 0.4
	H292	FNWYVDGVEVHNAKTKPR	4.9 ± 2.9	2.8 ± 2.9
	H294	TKPR	10.6 ± 7.6	19.3 ± 1.3
	H330	VSNKALPAPIEK	3.5 ± 0.3	2.5 ± 0.1
	H338	ALPAPIEKTISK	19.6 ± 2.1	22.6 ± 1.6
	H342	TISK	0.0 ± 0.0	0.1 ± 0.1
	H344	AKGQPR	4.4 ± 0.3	3.0 ± 0.8
	H396	GFYPSDIAVEWESNGQPENNYKTTPPV LDSDGSFFLYSK	0.2 ± 0.2	0.0 ± 0.0
	H418	LTVDKSR	10.4 ± 1.2	10.0 ± 1.0

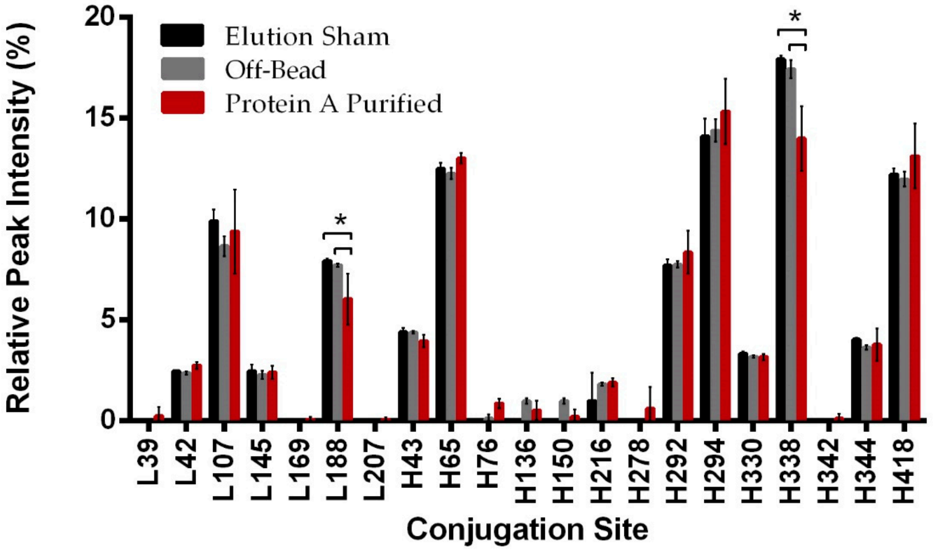

Figure S1: Relative peak intensity of conjugation sites as analyzed by LC/MS following tryptic digestion. All samples are T-DM1 Off-Bead ADCs from the same batch split into 3 groups. Off-Bead: No additional handling; Elution Sham: T-DM1 was exposed to elution buffer (50 mM glycine-HCl) for 5 minutes and then neutralized; Protein A Purified: T-DM1 ADCs were bound to Protein A magnetic beads to simulate a purification protocol then eluted and neutralized. Following treatment, all 3 groups underwent the peptide mapping digestion as described previously. LC/MS data of the digested peptides indicates statistically significant shifts in relative population between Protein A Purified and the other samples at 2 lysine conjugation sites as indicated by the *.

Table S2: Percent weighted spectral difference (%WSD) of Off-Bead and On-Bead relative to an unmodified trastuzumab antibody. Values are reported as average percent ± SD of triplicates.

Wavenumber Range (cm^{-1})	Off-Bead (%WSD)	On-Bead (%WSD)
823 – 836.5	2.75 ± 0.41	2.49 ± 0.57
849 – 816.5	0.49 ± 0.20	0.35 ± 0.11
1547.5 – 1561.5	1.14 ± 0.37	0.97 ± 0.07
1642.5 – 1670.5	9.74 ± 0.32	11.78 ± 0.71
1671 – 1697	3.89 ± 0.83	4.11 ± 0.89
